# Pramipexole inhibits astrocytic NLRP3 inflammasome activation via Drd3-dependent autophagy in a mouse model of Parkinson’s disease

**DOI:** 10.1038/s41401-022-00951-1

**Published:** 2022-07-27

**Authors:** An-qi Dong, Ya-ping Yang, Shu-min Jiang, Xiao-yu Yao, Di Qi, Cheng-jie Mao, Xiao-yu Cheng, Fen Wang, Li-fang Hu, Chun-feng Liu

**Affiliations:** 1 Department of Neurology and Clinical Research Center of Neurological Disease, The Second Affiliated Hospital of Soochow University, Suzhou, 215004 China; 2 Jiangsu Key Laboratory of Neuropsychiatric Diseases and Institute of Neuroscience, Soochow University, Suzhou, 215123 China; 3 Department of Neurology, The Second Affiliated Hospital of Xinjiang Medical University, Urumqi, 830000 China

**Keywords:** Parkinson’s disease, neuroinflammation, astrocytes, NLRP3 inflammasome, autophagy, dopamine D3 receptor

## Abstract

Inflammation is one of the pathogenic processes in Parkinson’s disease (PD). Dopamine receptor agonist pramipexole (PPX) is extensively used for PD treatment in clinics. A number of studies show that PPX exerts neuroprotection on dopaminergic (DA) neurons, but the molecular mechanisms underlying the protective effects of PPX on DA neurons are not fully elucidated. In the present study, we investigated whether PPX modulated PD-related neuroinflammation and underlying mechanisms. PD model was established in mice by bilateral striatum injection of lipopolyssaccharide (LPS). The mice were administered PPX (0.5 mg·kg^−1^·d^−1^, i.p.) 3 days before LPS injection, and for 3 or 21 days after surgery, respectively, for biochemical and histological analyses. We showed that PPX administration significantly alleviated the loss of DA neurons, and suppressed the astrocyte activation and levels of proinflammatory cytokine IL-1β in the substantia nigra of LPS-injected mice. Furthermore, PPX administration significantly decreased the expression of NLRP3 inflammasome-associated proteins, i.e., cleaved forms of caspase-1, IL-1β, and apoptosis-associated speck-like protein containing a caspase recruit domain (ASC) in the striatum. These results were validated in LPS+ATP-stimulated primary mouse astrocytes in vitro. Remarkably, we showed that PPX (100–400 μM) dose-dependently enhanced the autophagy activity in the astrocytes evidenced by the elevations in LC3-II and BECN1 protein expression, as well as the increase of GFP-LC3 puncta formation. The opposite effects of PPX on astrocytic NLRP3 inflammasome and autophagy were eliminated by *Drd3 depletion*. Moreover, we demonstrated that both pretreatment of astrocytes with autophagy inhibitor chloroquine (40 μM) in vitro and astrocyte-specific *Atg5 knockdown* in vivo blocked PPX-caused inhibition on NLRP3 inflammasome and protection against DA neuron damage. Altogether, this study demonstrates an anti-neuroinflammatory activity of PPX via a Drd3-dependent enhancement of autophagy activity in astrocytes, and reveals a new mechanism for the beneficial effect of PPX in PD therapy.

## Introduction

Parkinson’s disease (PD) is a common neurodegenerative disorder, affecting approximately 2% old population over 60. Patients with PD suffer from motor dysfunction (resting tremor, rigidity, and bradykinesia), and also non-motor symptoms (autonomic dysfunction, hyposmia, REM sleep behaviors, etc.) that occur years before the onset of motor phenotypes [1, 2]. The etiology and pathogenesis of PD remain elusive. It is pathologically featured by progressive losses of dopaminergic (DA) neurons in the substantia nigra pars compacta (SNpc) and the formation of α-synuclein-enriched Lewy bodies. The DA precursor levodopa is widely used for DA replacement and serves as the first-line treatment for PD [3]. However, this offers only symptomatic relief, neither curative nor protective, and is also associated with side effects and efficacy decay over time.

Direct stimulation of DA receptors (DRs) with their agonists represents an alternative strategy for PD therapy. Several DA agonists including pramipexole (PPX), ropinirole, and rotigotine are now approved and available for use in the clinic. These agonists are indicated for the symptomatic relief of early and advanced PD. They also have the advantage to reduce the dosage of levodopa and its associated motor fluctuations when used in combination [4]. Interestingly, DA agonist PPX shows efficacy in improving depressive symptoms in patients with PD [5]. PPX exerted neuroprotection on DA neurons, involving both receptor-dependent and -independent signaling [6–8]. DA modulates innate immunity and suppresses systemic inflammation via different subtypes of DRs [9]. DA also suppresses the neuroinflammation in the brain via astrocytic Drd2 and downstream signaling [10]. Neuroinflammation is implicated in DA neurodegeneration, serving as a key contributor to the onset and progression of PD [11–14]. All these indicate that PPX may affect PD-associated neuroinflammation and thus produce additional benefits for PD patients. Yet, the underlying cellular and molecular mechanisms are not fully elucidated. Previously, we demonstrated the impact of D2R/D3R agonist on macroautophagy and α-synuclein degradation in DA neurons and cell lines [15]. This prompted us to ask if PPX may modulate PD-related neuroinflammation via autophagic machinery.

In the present study, we demonstrated that PPX cotreatment attenuated the neuroinflammation and DA neuron losses in a PD mouse model induced by striatal lipopolyssaccharide (LPS) injection. More importantly, we found that PPX suppressed astrocytic NLRP3 inflammasome activation and IL-1β production in vivo and in vitro, but enhanced the astrocytic autophagy activity in a Drd3-dependent manner. Furthermore, pharmacologic and genetic repression of autophagy could abolish the effect of PPX on astrocytic NLRP3 inflammasome assembly and IL-1β secretion in vitro and in vivo. Altogether, the findings identify a novel mechanism for PPX-inhibited neuroinflammation in the brain, which was attributed to autophagy enhancement via stimulation of Drd3 in astrocytes.

## Materials and methods

### Reagents and antibodies

Pramipexole dihydrochloride (PPX, 4174, Tocris Bioscience, Bristol, UK) was purchased from Tocris Bioscience. LPS (055: B5, L6529, Sigma, St. Louis, MO, USA), adenosine 5′-triphosphate (ATP, A1852), chloroquine (CQ, C6628), and all other reagents unless specified were purchased from Sigma-Aldrich. The sources of primary antibodies were listed as follows: anti-LC3 (NB100-2220, NOVUS, Colorado, USA), anti-p62 (P0068, Sigma, St. Louis, MO, USA), anti-tyrosine hydroxylase (TH) (ab112, Abcam, Cambridge, UK) for Western blotting, anti-TH (T1299, Sigma, St. Louis, MO, USA) for immunohistochemistry study, anti-DAT (MAB369, Millipore, Darmstadt, Germany), anti-glial fibrillary acidic protein (GFAP) (130300, Thermo Fisher Scientific, MA, USA), anti-Iba1 (019-19741, Wako, Japan), anti-NeuN (MAB377, Chemicon), anti-NLRP3 (AG-20B-0014-C100, AdipoGen Life Sciences, San Diego, USA), anti-BECN1 (3738, Cell Signaling Technology (CST), MA, USA), anti-ULK1 (4773, CST, MA, USA), anti-Phospho-ULK (Ser555) (2449230, Millipore, MA, USA), anti-mTOR (2983, CST, MA, USA), anti-Phospho-mTOR (Ser2448) (2971, CST, MA, USA), anti-Atg5 (A2859, Sigma, USA), and β-actin (A3584, Sigma, MO, USA). The pro-IL-1β, cleaved IL-1β, pro-caspase-1 and cleaved caspase-1 blots were with different antibodies, anti-IL-1β (AF-401-NA, R&D Systems, Minnesota, USA), anti-cleaved IL-1β (Asp117) (52718, CST, MA, USA), anti-caspase-1 (14F468, Santa Cruz, Texas, USA), anti-cleaved caspase-1 (Asp296) (67314, CST, MA, USA) for cellular Western blotting, and anti-IL-1β (D6D6T) (31202, CST, MA, USA), anti-caspase-1 (E2Z1C) (24232, CST, MA, USA), and anti-ASC (67824, CST, MA, USA) for animal tissue study.

### Animals

Eight-week-old male C57BL/6 mice were purchased from Shanghai Laboratory Animal Center (Shanghai, China). *Atg5*^*flox/flox*^ mice were obtained from Japan RIKEN Laboratory Animal Center, kindly gifted by Prof. Noboru Mizushima (Tokyo, Japan). Genotyping was performed by polymerase chain reaction (PCR) according to the published protocol [16]. All mice were maintained under a 12 h light/12 h dark cycle with food and tap water *ad libitum*. All animal procedures were approved by Institutional Animal Care and Use Committee of Soochow University and complied with Soochow University laboratory animal care and use principles.

### Primary astrocytes culture

Primary astrocytes were obtained from P1–P3 neonatal C57BL/6J WT or *Drd3* knockout (KO) pups. *Drd3* KO mice were gifted by Prof Jia-wei Zhou (Institute of Neuroscience, Chinese Academy of Science, Shanghai, China). Briefly, cerebral cortices were dissected and the meninges were carefully stripped off. Tissues were then dissociated in 0.25% trypsin for 10 min at 37 °C and filtered through a 70 µm mesh. After centrifugation, cells were harvested and plated in 75 cm^2^ T-flask and maintained in Dulbecco’s modified Eagle’s medium (DMEM, 12430112, Gibco, USA) supplemented with 10% fetal bovine serum (30067334, Gibco, USA) and 1% penicillin/streptomycin. Culture medium was replaced with fresh DMEM 24 h later and changed every 3 days until reaching confluence. Floating microglia were removed by orbital shaking at 180 × *g* for 2 h, and then astrocytes at the lower layer were dissociated in 0.25% trypsin and harvested by centrifuge at 1000 × *g* for 10 min. The pellets were gently dispersed and plated on 12-well plates for experimentation further.

### Quantitative real-time PCR (qPCR)

Total RNA was extracted using Trizol reagent (15596018, Invitrogen, MA, USA) and reverse transcribed into cDNA with a cDNA synthesis kit (K1622, Thermo Fisher Scientific, MA, USA). Quantitative PCR was performed with SYBR-Green premix Ex Taq (A25742, Thermo Fisher Scientific, MA, USA) and detected by 7500 Real-Time PCR System (Applied Biosystems, Foster City, CA, USA). 18S served as an internal control gene. qPCR primers were synthesized by Genscript Biotech Corporation (Nanjing, China) and the sequences are listed in Table 1.

**Table 1:** Primer sequences for quantitative real-time PCR.

*IL-1β*	5′-TGGAAAAGCGGTTTGTCTT-3′ (forward)
	5′-TACCAGTTGGGGAACTCTGC-3′ (reverse)
*TNF-α*	5′-CATCTTCTCAAAATTCGAGTGACAA-3′ (forward)
	5′-TGGGAGTAGACAAGGTACAACCC-3′ (reverse)
*IL-6*	5′-GAGGATACCACTCCCAACAGACC-3′ (forward)
	5′-AAGTGCATCATCGTTGTTCATACA-3′ (reverse)
*iNOS*	5′-CAGGAGGAGAGAGATCCGATTTA-3′ (forward)
	5′-GCATTAGCATGGAAGCAAAGA-3′ (reverse)
*Drd3*	5′-GAACTCCTTAAGCCCCACCAT-3′ (forward)
	5′-GAAGGCCCCGAGCACAAT-3′ (reverse)
*Drd2*	5′-TGGACTCAACAACACAGACCAGAATG-3′ (forward)
	5′-GATATAGACCAGCAGGGTGACGATGA-3′ (reverse)
*18S*	5′-TCAACACGGGAAACCTCAC-3′ (forward)
	5′-CGCTCCACCAACTAAGAA C-3′ (reverse)

### Cytokine measurement

The cytokine contents in cell culture supernatants and mouse brain homogenates were measured by Enzyme-Linked-Immunosorbent-Assay (ELISA) kits according to the manufacturers’ instructions. The ELISA kits for IL-1β (88-7013-88) and tumor necrosis factor-alpha (TNF-α) (88-7324-77) assay were purchased from Thermo Fisher Scientific while that for interleukin-10 (IL-10) (M1000B) was from R&D Systems.

### Cell viability assay

Cell viability was evaluated using a cell counting kit-8 (CCK-8) (HY-K0301, MCE, Shanghai, China). In brief, cells were cultured in a 96-well plate and treated with PPX at various concentrations for 12 h. At the end of treatment, 10 μL CCK-8 solution was added to each well and incubated at 37 °C for 1 h. After that, the optical densities were measured by a microplate reader at a wavelength of 450 nm.

### Western blotting

Cells and tissues were lysed in chilled RIPA buffer (P0013B, Beyotime, Shanghai, China) with cocktails of protease (B14001, Biomark, Houston, USA) and phosphatase inhibitor (HY-K0022, MCE, Shanghai, China). After centrifugation at 12,000 × *g* for 30 min, the supernatant was harvested and the protein concentration was determined using a BCA assay kit (23227, Thermo Scientific, MA, USA). The lysates were solubilized in sodium dodecyl sulfate (SDS) sample buffer and separated by 12% SDS-polyacrylamide gel electrophoresis. Next, proteins were transferred to polyvinylidene fluoride membrane and blocked with 5% non-fat milk powder in 0.1% Tris-buffered saline with 0.05% Tween-20 (TBST) for 2 h at room temperature (RT). After that, membranes were incubated with primary antibodies overnight at 4 °C, rinsed in TBST and then incubated with the corresponding secondary antibody (1:5000; Jackson Immuno Research Laboratories) for 1 h at RT. Finally, the blots were rinsed in TBST and visualized using a chemiluminescence kit (P10300, New Cell & Molecular Biotech, Shanghai, China). Densitometric analysis was performed using ImageJ software.

### Immunohistochemistry study

For histological evaluations, mice were anesthetized and transcardially perfused with ice-cold saline followed by 4% paraformaldehyde (PFA). The brains were postfixed in 4% PFA overnight and immersed into a serial of a 10%–30% sucrose solution for dehydration. A series of coronal sections (20 µm) were cut with a cryostat (Leica, Wetzlar, Germany). For 3,3′-diaminobenzidine (DAB) staining, the sections were treated with 3% H_2_O_2_ for 10 min and then blocked with 2% bovine serum albumin (BSA) and 0.5% Triton X-100 in PBS for 1 h at RT. After that, sections were incubated with anti-TH (1:500, Sigma, T1299) at 4 °C overnight. Next, sections were incubated with HRP-conjugated secondary antibodies for 1 h and visualized using a DAB solution (GK500705, Genentech, Shanghai, China). For fluorescence immunohistochemistry, the sections were blocked in 5% BSA for 1 h and incubated with primary antibodies at 4 °C overnight followed by secondary antibodies tagged with Alexa 488 (green) or 555 (red) (Vector Labs, California, USA). Nuclei were stained with 4′,6-diamidino-2-phenylindole dihydrochloride (DAPI, H-2000-2, Vector Labs, California, USA). Images were obtained under a bright field or fluorescence microscope with Zen 2011 software (Zeiss, LSM 780, Carl Zeiss, Jena, Germany).

### GFP-LC3 dots quantification

The impact of PPX on astrocyte autophagy was evaluated by GFP-LC3 dots assay as previously reported [15]. In brief, astrocytes were transiently transfected with GFP-LC3B plasmids (25185, Addgene, MA, USA) using Lipofectamine 2000 (12566014, Invitrogen, MA, USA). Thirty-six hours post-transfection, cells were treated with PPX for 12 h. After that, cells were observed under confocal microscopy (Zeiss, LSM700) and the number of GFP-LC3 dots formed per cell was counted, with at least 20 cells included for analysis for each group.

### Transmission electron microscopy

Cells were pre-fixed with ice-cold 2.5% glutaraldehyde in 0.1 M phosphate-buffered saline (pH 7.4), and postfixed with 1% osmium tetroxide buffer for 2 h. After dehydration in a gradient series of ethyl alcohol, cells were embedded in epoxy resin. Ultrathin sections were stained with uranyl acetate and lead citrate, and examined using a transmission electron microscope (JEM 1400, JOEL, Tokyo, Japan).

### *Sholl* analysis for glial cell morphology

We performed *Sholl* analysis for glial cell morphology as described previously [17]. In brief, images at high resolutions were obtained with OLYMPUS VS200 after immunofluorescent staining. *Sholl* analysis was performed with the plugin of *Sholl* analysis in ImageJ that automatically draws a series of concentric circles at 10-μm intervals from the center of DAPI signal to the end of the most distant process in every single cell. The number of intercepts of GFAP or ionized calcium-binding adapter molecule (Iba1) positive processes in each circle, ending radius, and ramification index were analyzed. A total of 20 cells were analyzed in each group.

### LPS-induced PD model and drug treatment

LPS-induced PD model was performed as described previously [18]. Mice were randomly grouped, anesthetized, and fixed in a stereotaxis instrument. LPS (5 μg dissolved in 1 μL saline) was injected into the bilateral striatum (1 μL/site) using a 5 μL Hamilton syringe. The coordinates relative to bregma were: anteroposterior (AP) +1.18 mm, mediolateral (ML) ±1.5 mm, dorsoventral (DV) –3.5 mm; AP: –0.34 mm, ML: ±2.5 mm, DV: –3.2 mm. After surgery, mice were kept on a warm pad until recovery. For PPX treatment, mice were intraperitoneally treated with PPX (0.5 mg·kg^−1^·d^−1^, i.p.) or sterilized saline 3 days before stereotaxic surgery and 3 or 21 days after surgery for biochemical and histological study, respectively.

### Astrocyte-specific *Atg5* knockdown in vivo

To induce astrocyte-specific *Atg5* deficiency in vivo, adeno-associated virus (AAV) AAV2/8-short-GFAP-EGFP-T2A-Cre (AAV-eGFP-Cre) (OBiO Biotechnology, Shanghai, China) was stereotaxically injected into the striatum of adult *Atg5*^*flox*/*flox*^ mice at a volume of 4 µL 3.11 × 10^12^ VG/mL, with the coordinates at: AP +0.4 mm, ML ±2.0 mm, DV –3.5 mm (relative to the bregma). For control group, AAV2/8-short-GFAP-MCS-EGFP-3FLAG (AAV-control) was given.

### Statistical analysis

All the data were presented as mean ± SEM. Statistics were performed using GraphPad Prism software. The differences were analyzed using Student’s *t*-test for two-group comparison or one-way analysis of variance (ANOVA) for multiple-group comparison followed by Tukey post hoc analysis. Differences were considered significant at values of *P* < 0.05.

## Results

### PPX attenuated the DA neuron damage in the SN caused by striatal LPS injection

**Fig. 1:**
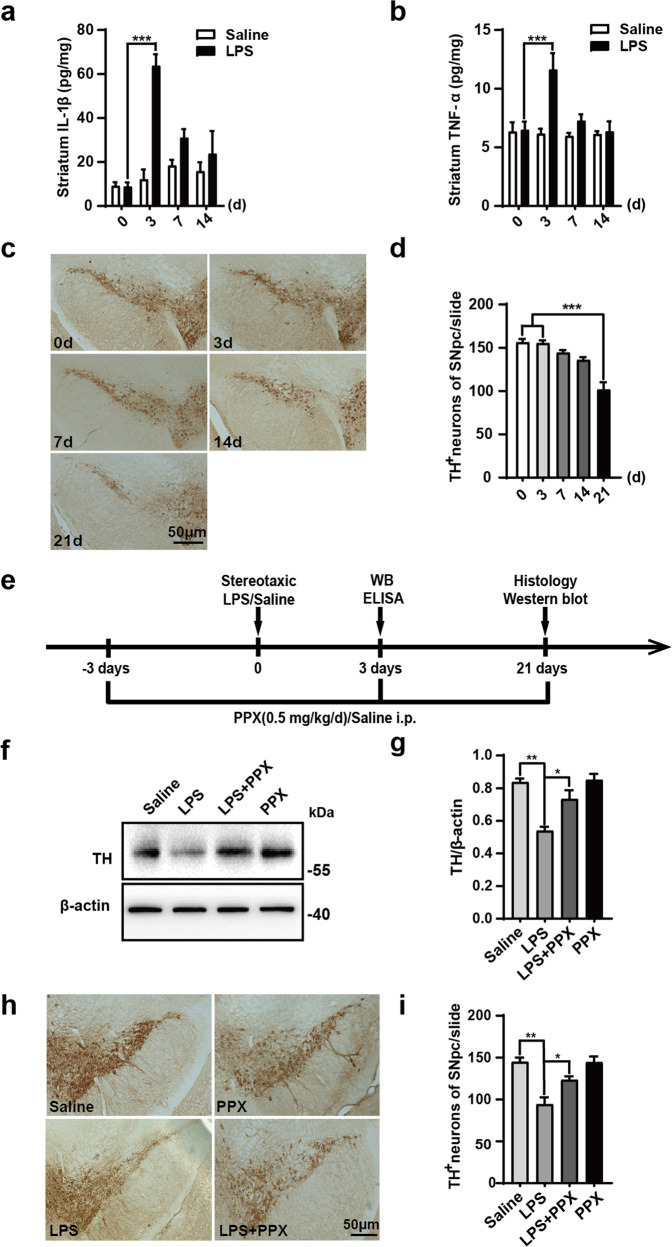
Striatal LPS injection caused neuroinflammation and DA neuron losses in the substantia nigra. **a**, **b** ELISA assays for the striatal IL-1β (**a**, *n* = 4) and TNF-α (**b**, *n* = 5) levels in LPS or saline-injected mice at 0, 3, 7, and 14 days. **c**, **d** Immunostaining (**c**) and quantification (**d**) of TH-positive neurons in the mice SNpc on different days after LPS injection. Scale bar: 50 μm. *n* = 3 mice per group. **e** Experimental flow chart. C57BL/6 mice were intraperitoneally treated with PPX (0.5 mg·kg^−1^·d^−1^) or saline 3 days before and 21 days after the stereotaxic injection of LPS or saline into the striatum. ELISA and Western blotting for neuroinflammatory responses and immunostaining for TH-positive neurons quantification were performed at 3 and 21 days after LPS injection, respectively. **f**, **g** Western blots (**f**) and group data (**g**) for striatal TH protein levels, *n* = 3 per group. **h**, **i** Representative images for immunostaining with anti-TH (**h**) and number of TH^+^ neurons in the SNpc (**i**). Scale bar: 50 μm. *n* = 3 mice per group. One-way ANOVA followed by Tukey’s post hoc analysis. Mean ± SEM. *P < 0.05, **P < 0.01, ***P < 0.001 vs. as indicated.

Neuroinflammation plays an indispensable role in PD progression. To explore the effect of dopamine receptor (Drd2/3) agonist PPX on neuroinflammation and DA neuron damage, we established a neuroinflammation-related PD mouse model using a stereotaxic injection of LPS into the striatum. The striatal accumulation of proinflammatory cytokines was determined via ELISA in different periods after LPS injection. The results showed that IL-1β and TNF-α levels dramatically increased at 3 days after LPS injection compared to the saline group, and gradually returned to the basal level at 7 and 14 days after surgery (Fig. 1a, b). The number of TH (a DA neuron marker) positive neurons in the SNpc appeared to decline in a time-dependent manner, which dropped about 30% of the basal level at 21 days after injection (Fig. 1c, d). The LPS-caused DA neuron losses, as evaluated by striatal TH protein level and SN TH^+^ neuron counting, were obviously attenuated by PPX administration, which was systemically given at 3 days before LPS injection and continued for 21 days after surgery (Fig. 1e–i).

### PPX suppressed astrocytes activation and neuroinflammation in the striatum of LPS-injected mice

**Fig. 2:**
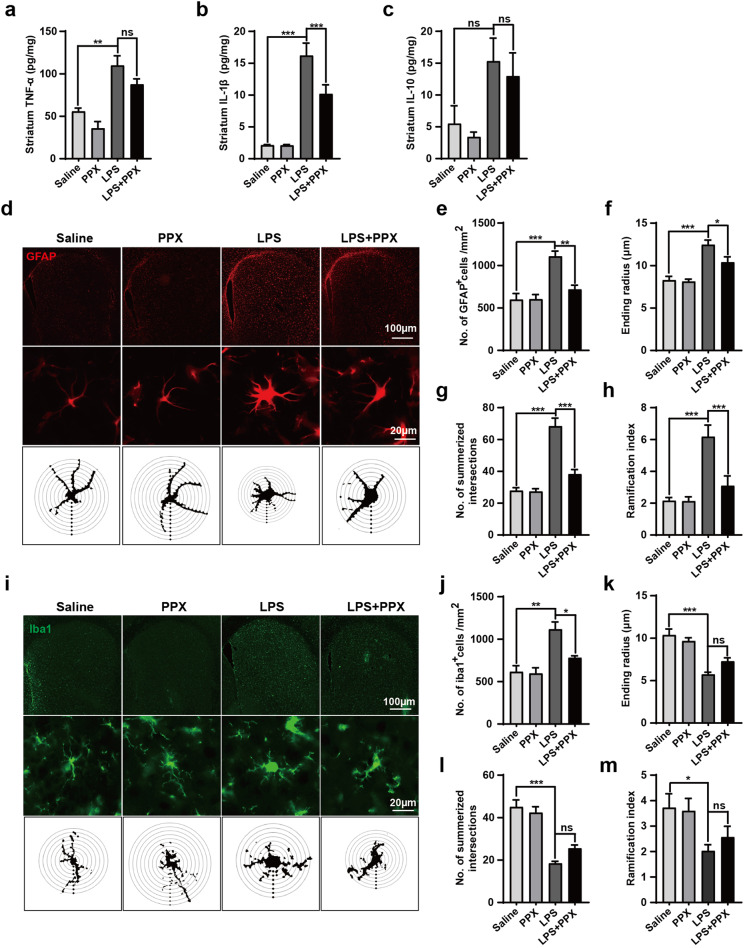
PPX administration attenuated astrocyte activation and IL-1β generation in the striatum of LPS-injected mice. **a**–**c** ELISA results for TNF-α (**a**, *n* = 5), IL-1β (**b**, *n* = 6), and IL-10 (**c**, *n* = 5) levels in the striatum of mice. **d**–**h** Immunofluorescent anti-GFAP staining for studying astrocyte activation/proliferation in the striatum. GFAP^+^ cells quantification shown in **e**, *n* = 4–6 mice per group. The lower panel in **d** showed a representative GFAP^+^ cell morphology generated by Sholl analysis, and group data for ending radius (**f**), summarized intersects (**g**) and ramification index (**h**) are shown in **f**–**h**, *n* = 20 cells per group for analysis. The interval of the concentric circles is 10 μm. Scale bar: 100 μm (upper panel) and 20 μm (lower panel). **i**–**m** Immunofluorescent staining with anti-Iba1 to assess microglia activation in the striatum and microglia morphological changes by Sholl analysis (*n* = 20 cells per group), with 5 μm intervals for the concentric circles. Iba1^+^ cells quantification results are shown in **j**–**m**. One-way ANOVA followed by Tukey’s post hoc analysis. Mean ± SEM. *P < 0.05, **P < 0.01, ***P < 0.001 vs. as indicated; ns, not significant.

We then studied if PPX affected the neuroinflammatory responses in this mouse model. ELISA results revealed that the striatal proinflammatory cytokines IL-1β and TNF-α were significantly enhanced in LPS-injected mice while the anti-inflammatory IL-10 showed a tendency but not a significant increase. Notably, PPX cotreatment reduced the accumulation of IL-1β but not TNF-α or IL-10 in the striatum (Fig. 2a–c). Immunofluorescent staining, in combination with Sholl analysis for glial cell morphology, displayed that LPS injection caused a marked effect on the proliferation and activation of glial cells in the striatum. Specifically, the number of both astrocytes (GFAP^+^) and microglia (Iba1^+^) was enhanced in LPS-injected mice compared to saline-injected controls. Compared with LPS-injected mice, PPX cotreatment group showed significant decreases in the number of GFAP^+^ astrocytes and their morphological changes (number of intersections, ending radius, and ramification index) (Fig. 2d–h). Meanwhile, PPX cotreatment reduced the number of Iba1^+^ microglia in LPS-injected mice; however, it merely showed a mild but insignificant impact on microglia morphology (Fig. 2i–m). These data indicated that PPX had a predominant effect on astrocyte activation and IL-1β generation, ameliorating the neuroinflammatory responses caused by striatal LPS injection.

### PPX inhibited the astrocytic NLRP3 inflammasome activation in vivo and in vitro

**Fig. 3:**
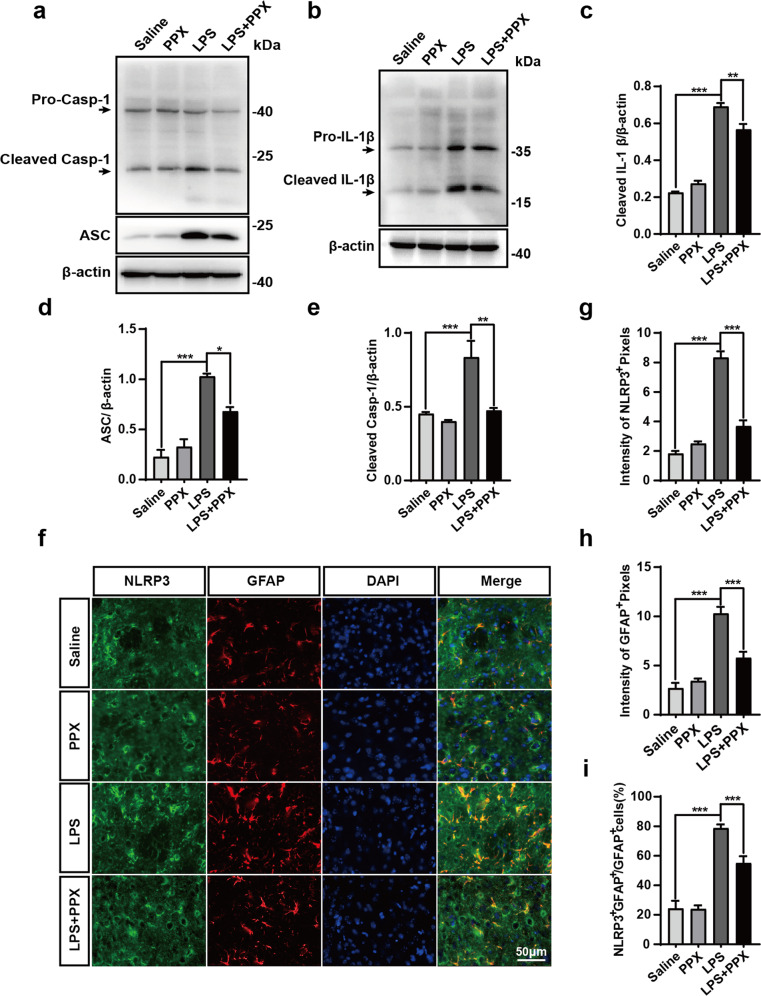
PPX cotreatment ameliorated striatal astrocytic NLRP3 inflammasome activation in mice. **a**–**e** Western blots (**a**, **b**) and group data of cleaved IL-1β (**c**), ASC (**d**), and cleaved Casp-1 (**e**) protein levels in the striatum, *n* = 3. **f**–**i** Representative images (**f**) showing NLRP3^+^ (green) and GFAP^+^ (red) fluorescent signals, scale bar is 50 μm. Quantification for NLRP3^+^ (**g**, *n* = 4) and GFAP^+^ (**h**, *n* = 4) intensities and the percentage of NLRP3^+^GFAP^+^ over GFAP^+^ cells (**i**, *n* = 4), assessed by ImageJ software. One-way ANOVA followed by Tukey’s post hoc analysis. Mean ± SEM. *P < 0.05, **P < 0.01, ***P < 0.001 vs. as indicated. Casp-1 caspase-1.

NLRP3 inflammasome is an intracellular protein complex that regulates caspase-1 activity and IL-1β generation. A great body of evidence suggests a role of NLRP3 inflammasome in PD pathogenesis and indicates that suppression of NLRP3 inflammasome activation can alleviate DA neuron injury [19–22]. As PPX exhibited a preferable inhibition on IL-1β production induced by striatal LPS injection (Fig. 2a), we speculated that PPX may have an effect on NLRP3 inflammasome activity. To test this hypothesis, total and cleaved forms of several inflammasome-associated protein levels in the striatum were examined. Western blot revealed a remarkable increase in cleaved forms of caspase-1 and IL-1β, as well as ASC protein levels in LPS-treated group, and these enhancements were attenuated in PPX cotreated group (Fig. 3a–e). Immunostaining displayed a lower immunoreactivity of NLRP3 in PPX+LPS group relative to LPS group (Fig. 3f, g). GFAP fluorescence intensity was also decreased in PPX-cotreated group, which was consistent with GFAP^+^ cell quantification results in Fig. 2e. In addition, an obvious co-localization of NLRP3^+^ fluorescence to GFAP^+^ cells was found in the striatum of LPS-treated mice, and the ratio of double-positive (NLRP3^+^GFAP^+^) over GFAP^+^ cells was reduced in PPX-cotreated group (Fig. 3i). This implicated that PPX was able to suppress astrocytic NLRP3 inflammasome assembly and activation caused by striatal LPS injection in mice.

**Fig. 4:**
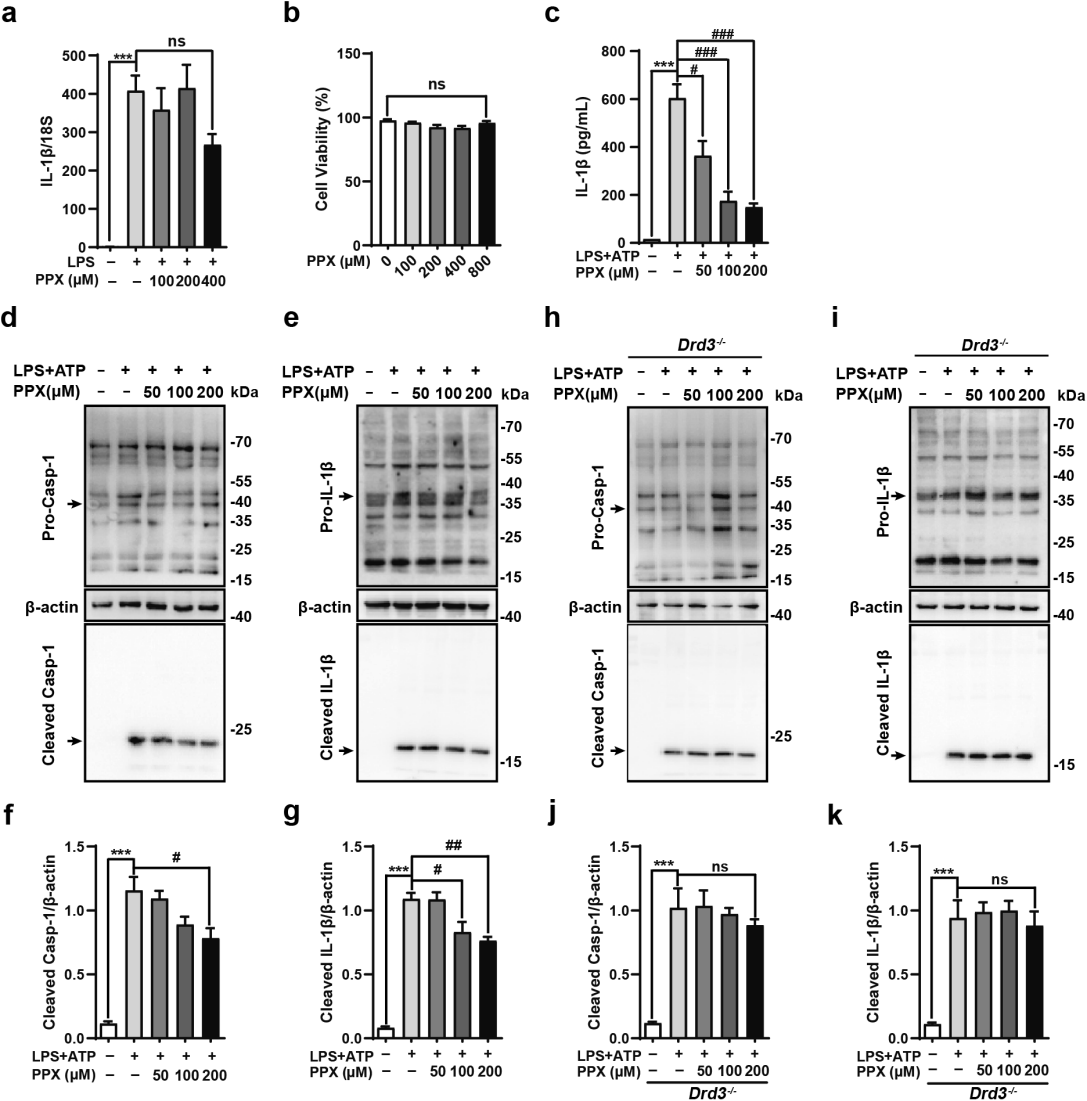
PPX suppressed ATP-induced inflammasome activation in cultured astrocytes. **a** PPX did not affect IL-1β mRNA levels induced by LPS in primary cultured astrocytes, 18S serving as internal controls. Cells were pretreated with PPX (100, 200, and 400 μM) for 12 h, followed by LPS (1 μg/mL) stimulation for additional 6 h. *n* = 4, ***P < 0.001, vs. control group; ns, not significant. **b** Effect of PPX treatment for 12 h on the cell viability of astrocytes, *n* = 4. ns, not significant. **c** ELISA results for supernatant IL-1β level in astrocytes treated as in **d**, **e**. *n* = 3, ***P < 0.001 vs. control group; ^#^*P* < 0.05, ^###^*P* < 0.001 vs. LPS + ATP group. **d**–**g** Western blot study for Casp-1 (**d**) and IL-1β (**e**) cleavage and maturation. Astrocytes were primed with 1 μg/mL LPS for 5 h, followed by PPX (50, 100, 200 μM) treatment for 1 h and subsequent ATP (5 mM) stimulation for 30 min. Densitometric analysis was shown in (**f**) and (**g**), *n* = 3. ***P < 0.001 vs. control group; ^#^*P* < 0.05, ^##^*P* < 0.01 vs. LPS + PPX group. **h**–**k** Western blotting (**h** and **i**) and quantification of Casp-1 (**j**) and IL-1β (**k**) cleavage in *Drd3*^*−/−*^ astrocytes treated as in **d**, **e**, *n* = 3. Mean ± SEM. ***P < 0.001 vs. control group; ns, not significant. One-way ANOVA followed by Tukey’s post hoc analysis. Casp-1 caspase-1.

The inhibitory effect of PPX on astrocytic NLRP3 inflammasome was verified in vitro. Quantitative PCR revealed that PPX, at the concentration range from 100 to 400 μM, did not alter the increase of IL-1β transcription induced by LPS (Fig. 4a), nor the cell viability of primary astrocytes (Fig. 4b). However, PPX pretreatment (50, 100, 200 μM) was able to reduce the supernatant IL-1β level in ATP-stimulated LPS-primed astrocytes (Fig. 4c). Moreover, in the presence of PPX cotreatment, cleaved forms of caspase-1 and IL-1β protein levels were decreased compared to ATP-stimulated primed cells (Fig. 4d–g). PPX is a selective agonist for dopamine D2-like receptor, with a higher affinity to Drd3 over Drd2 [23]. To clarify if Drd3 was responsible for the inhibition of PPX on astrocytic NLRP3 inflammasome, we prepared primary astrocyte cultures from neonatal *Drd3* KO (*Drd3*^*−/−*^) mice. Western blot study showed no alterations in cleaved caspase-1 or IL-1β protein levels in ATP-stimulated primed *Drd3 depleted* astrocytes, even in the presence of PPX cotreatment at various concentrations (Fig. 4h–k), indicating that Drd3 is required for the restriction of PPX on astrocytic inflammasome activation.

### PPX enhanced the autophagy activity in astrocytes

**Fig. 5:**
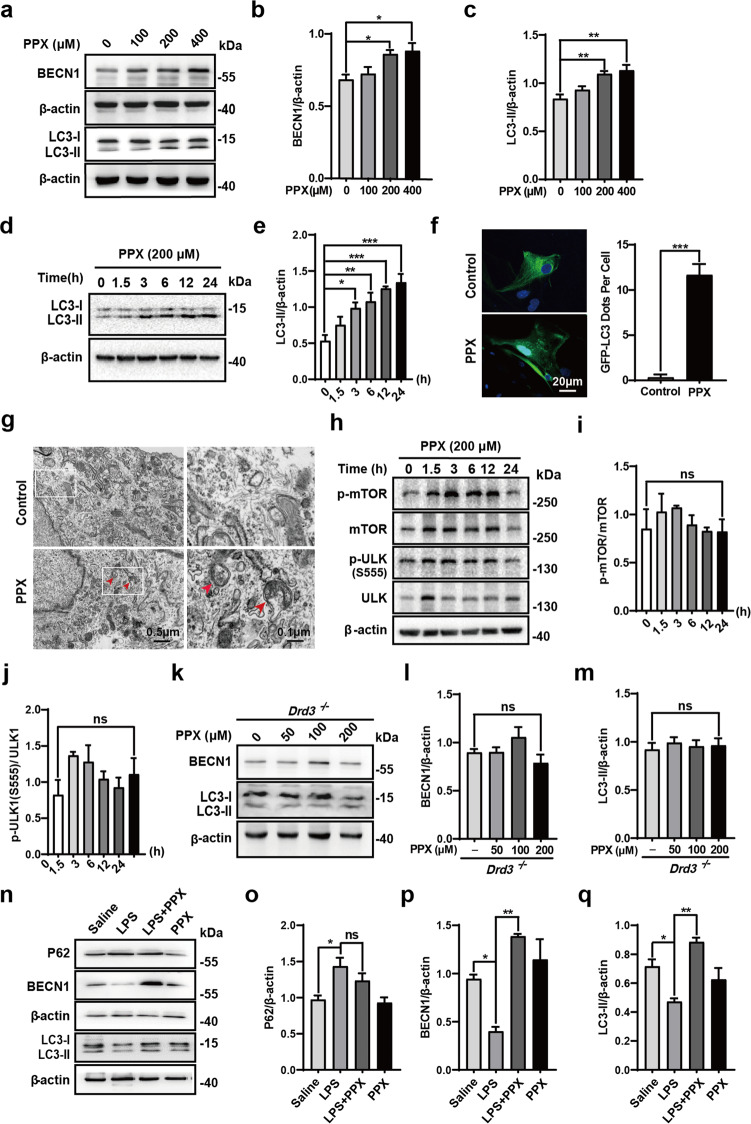
PPX enhanced the autophagy activity in astrocytes via Drd3-dependent signaling. **a**–**c** Dose-dependent effects of PPX on BECN1 (**b**) and LC3-II (**c**) expressions in astrocytes, *n* = 3. One-way ANOVA followed by Tukey’s post hoc analysis. *P < 0.05, **P < 0.01 vs. control group; ns, not significant. **d**, **e** Time profile of PPX (200 μM) treatment on LC3-II level. *n* = 3. *P < 0.05, **P < 0.01, ***P < 0.001 vs. control group. **f** Representative images and quantification for GFP-LC3 puncta. Astrocytes were transfected with GFP-LC3 plasmid for 48 h, and then treated with PPX or PBS for 12 h. At least 20 cells per group were included for analysis. Scale bar: 20 μm. ***P < 0.001 vs. control group, Student’s *t*-test. **g** Representative TEM images for treated with PBS or PPX-treated primary astrocytes. Double-membraned vesicles (red arrows) were observed in PPX group and the corresponding region in the white box is magnified in the right panel. **h**–**j** Time profile of PPX treatment on phosphorylation of mTOR (S2248) and ULK1 (S555), as well as their total levels in astrocytes, *n* = 3. ns, not significant. **k**–**m** Western blotting for BECN1 (**l**) and LC3-II (**m**) levels in *Drd3*^*−/−*^ astrocytes treated with PPX at diverse concentrations, *n* = 4. **n**–**q** Western blots for P62, BECN1 and LC3-II expressions in the striatum from LPS or saline-injected mice w/o PPX treatment. Densitometric analysis was shown in **o**–**q**, *n* = 3. Mean ± SEM. *P < 0.05, **P < 0.01 vs. as indicated; ns, not significant. One-way ANOVA followed by Tukey’s post hoc analysis.

To study the mechanism by which PPX restricted NLRP3 inflammasome activity in astrocytes, we examined the autophagy activity because a negative effect of autophagy on inflammasome assembly and activation was reported previously [24]. Western blot analysis revealed the increases of BECN1 and LC3-II protein levels in primary astrocytes following PPX (200, 400 μM) treatment for 24 h (Fig. 5a–c). A time-dependent increase of LC3-II protein was also found in PPX-treated cells (Fig. 5d, e). Consistently, an increased formation of GFP-LC3B puncta was found in PPX-treated astrocytes compared to controls (Fig. 5f). Transmission electron microscopy also observed a few double-membraned vesicles typical of autophagosomes in PPX-treated cells while those were rarely identified in the control group (Fig. 5g). Phosphorylations in mTOR (S2248) or ULK1 (S555) were not altered after PPX treatment (Fig. 5h–j), implying a less likely of mTOR involvement in the autophagy regulation by PPX in astrocytes. Notably, PPX did not alter LC3-II or BECN1 protein levels in *Drd3*^*−/−*^ astrocytes (Fig. 5k–m), in line with its effect on inflammasome activation (Fig. 4). These data suggest that PPX has the ability to enhance astrocytic autophagy activity via Drd3-dependent signaling. We further examined the protein levels of P62, BECN1, and LC3 in the striatum of LPS-injected mice with or without PPX cotreatment. As shown in Fig. 5n–q, P62 protein level was increased, while BECN1 and LC3-II levels were decreased in LPS-injected mice compared with the saline group. In PPX-cotreated group, the protein levels of BECN1 and LC3-II were significantly higher than those in LPS group, and P62 expression showed a tendency to decline, indicating that PPX treatment caused an enhancement of autophagy activity in the striatum of LPS-injected mice.

### Astrocytic autophagy deficiency abolished the inflammasome suppression by PPX

**Fig. 6:**
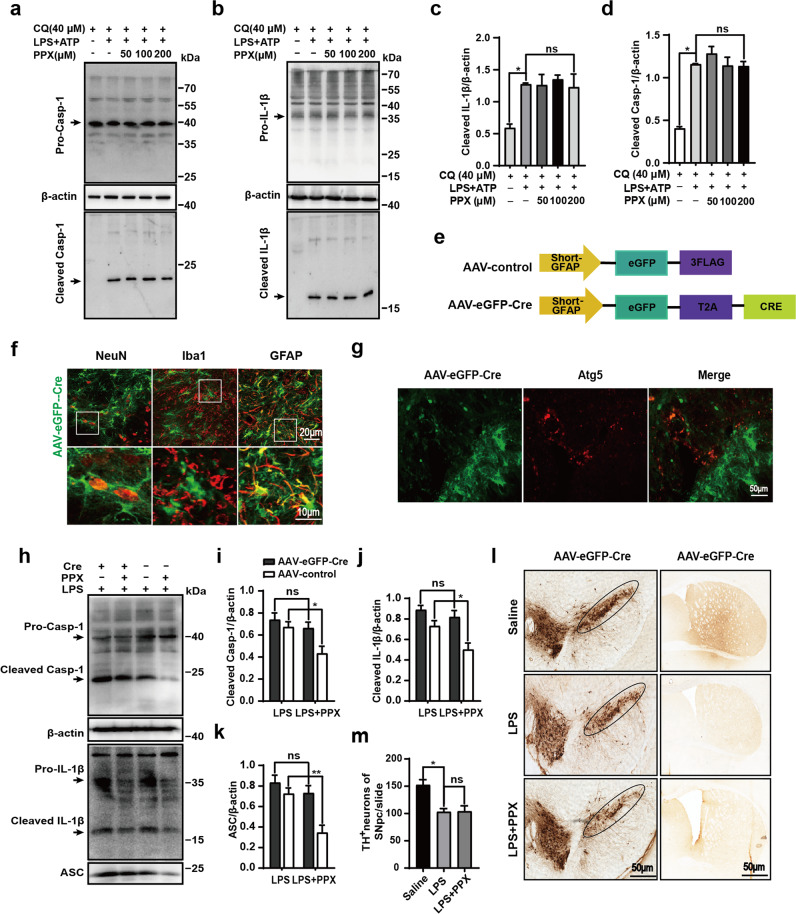
Astrocytic autophagy deficiency prevented the inhibitory effect of PPX on NLRP3 inflammasome activation in vivo and in vitro. **a**–**d** Astrocytes were incubated with lysosome inhibitor CQ (40 μM) for 2 h, and then subjected to treatments as in Fig. 4d–g. Analysis of Casp-1 (**a**) and IL-1β (**b**) cleavage was determined by Western blotting, with group data shown in **c** (cleaved IL-1β) and **d** (cleaved Casp-1). *n* = 3, One-way ANOVA followed by Tukey’s post hoc analysis. *P < 0.05 vs. as indicated; ns, not significant. **e** A simplified schematic diagram of AAV-control or AAV-eGFP-Cre structure. **f** Representative images showing a predominant localization of AAV-eGFP-Cre to GFAP^+^ but not Iba1^+^ or NeuN^+^ cells in the striatum of AAV-eGFP-Cre injected C57BL/6 mice at 3 weeks. Scale bar: 20 μm for the upper panel, and 10 μm for lower panel. **g** Atg5 immunoreactive signal (red) was almost undetectable in AAV-eGFP-Cre infected cells. Scale bar: 50 μm. *n* = 3. **h**–**k** Western blots (**h**) and group data of cleaved Casp-1 (**i**), cleaved IL-1β (**j**), and ASC (**k**) protein levels in the striatum. *n* = 4, two-way ANOVA. *P < 0.05, **P < 0.01 vs. as indicated; ns, not significant. One-way ANOVA followed by Tukey’s post hoc analysis. **l**, **m** Representative images for SNpc (left panel) and striatum (right panel) regions (**l**) and quantification of TH^+^ neuron in the SNpc (**m**). The striatum of *Atg5*^*flox/flox*^ mice was injected with AAV-eGFP-Cre 3 weeks before saline or LPS stereotaxic injection, and PPX was intraperitoneally administered 3 days before and 21 days after LPS injection as Fig. 1e. Mean ± SEM. *P < 0.05 vs. Saline group; ns, not significant. Scale bar: 50 μm. Casp-1 caspase-1.

Finally, we studied whether PPX-elicited inflammasome inhibition was autophagy dependent. To clarify this, we examined the levels of NLRP3 inflammasome-associated proteins in PPX-treated astrocytes with or without autophagy inhibition. Western blotting showed that PPX failed to reduce the cleaved caspase-1 and IL-1β protein levels induced by ATP and LPS priming in astrocytes in the presence of lysosomal inhibitor CQ, implying the requirement of autophagy in PPX modulated inflammasome activation (Fig. 6a–d). This was confirmed by in vivo study in astrocyte-specific *autophagy-related gene 5* (*Atg5)*-knockdown mice. A Cre-carrying AAV driven by the promoter GFAP (AAV-eGFP-Cre) was injected into the dorsal striatum of *Atg5*^*flox/flox*^ mice. For parallel controls, the mice were injected with AAV-eGFP driven by GFAP (AAV-control) (Fig. 6e). As expected, AAV-eGFP-Cre was highly expressed at 3 weeks after injection and predominantly co-localized to GFAP^+^ astrocytes, but not NeuN^+^ neurons or Iba1^+^ microglia, suggesting AAV-eGFP-Cre specifically infected astrocytes (Fig. 6f). In addition, there was almost no Atg5 immunoreactive fluorescence in astrocytes expressing AAV-eGFP-Cre (Fig. 6g), implying the successful establishment of astrocytic *Atg5 depletion* in mice. We then studied the effect of PPX treatment on LPS-induced inflammasome protein levels in astrocyte-specific *Atg5*-deficient mice by Western blotting. In AAV-control treated *Atg5*^*flox/flox*^ mice, LPS injection significantly enhanced the levels of cleaved caspase-1, IL-1β, and ASC proteins in the striatum, which was reduced by PPX administration. By contrast, the protein levels of these inflammasome components in PPX-cotreated AAV-eGFP-Cre mice were comparable to those in LPS-injected groups without PPX administration. There was no significant difference between these two groups (Fig. 6h–k). Subsequently, we tested whether astrocyte *Atg5 deficiency* had any effect on DA neuron losses. Immunostaining with anti‐TH and anti-DAT in the midbrain slices showed a marked loss of DA neurons and fibers in the SNpc and striatum of LPS-injected mice. However, PPX treatment did not alleviate the loss of DA neurons and DAT density caused by LPS in AAV-eGFP-Cre injected *Atg5*^*flox/flox*^ mice, suggesting that astrocytic *Atg5* deletion abolished the protective effect of PPX on DA neurons (Fig. 6l, m). Hence, PPX-elicited NLRP3 inflammasome suppression and protection against DA neuron damage was, at least in part, mediated by astrocytic autophagy activation.

## Discussion

PPX has been shown to mitigate both motor and non-motor symptoms in patients with PD [25, 26]. However, the underlying cellular and molecular mechanism remains to be investigated further. In the present study, we demonstrated that pretreatment with the Drd2/Drd3 agonist PPX attenuated the neuroinflammation and DA neuron losses caused by LPS injection. Moreover, PPX pretreatment showed a more pronounced inhibition of astrocyte activation and IL-1β production, only with a mild effect on microglia and TNF-α generation in the striatum. In vitro and in vivo studies further revealed an inhibition of PPX on astrocytic NLRP3 inflammasome activation, as well as IL-1β maturation and secretion in a Drd3-dependent manner. Lastly, the results identified a stimulatory effect of PPX on autophagy flux in astrocytes. More importantly, pharmacologic and genetic suppression of astrocytic *Atg5* gene abolished the impact of PPX on NLRP3 inflammasome assembly and activation, as well as the protective effect of PPX against DA neuron damage induced by LPS. Taken together, our findings revealed a novel mechanism for the inhibitory effect of PPX on neuroinflammation in the striatum, which was attributed to autophagy enhancement via stimulation of Drd3 in astrocytes.

Microglia and astrocytes play essential but different roles in neuroinflammation. Microglia sense the environmental cues and inflammatory stimuli and initiate the immune response while astrocytes communicate with microglia and regulate the neuroinflammatory responses depending on the context [27–29]. In this study, we observed a rapid reaction of both microglia and astrocytes which proliferated and transformed into activated morphologies early 3 days after LPS injection. However, PPX showed different effects on them. Although PPX pretreatment reduced the proliferation of both glial cells, it only repressed the astrocytic morphological changes, with a mild effect on microglia. This may be related to the properties of PPX, a non-ergot agonist of D_2_-like receptor family which includes Drd2, Drd3, and Drd4 subtypes. Compared with Drd2 (*K*_i_ = 3.9 nM) and Drd4 (*K*_i_ = 5.1 nM), the affinity of PPX to Drd3 (*K*_i_ = 0.5 nM) is the highest [4]. Besides, the expression profile of Drd3 seems to be cell specific. Drd3 was selectively expressed in astrocytes but not microglia both at transcription and protein levels [30, 31]. Relative to Drd2, Drd3 is predominantly expressed in the ventral striatum and other limbic brain regions, and is thought to mediate behavioral and motor complications in PD [32]. Therefore, the astrocytic Drd3 may be responsible for the inhibitory effect of PPX on NLRP3 inflammasome activation. This is supported by the in vitro data that PPX failed to suppress the inflammasome activity or enhance autophagy flux in *Drd3*^−/−^ astrocytes. Consistently, our previous study also revealed a role of Drd3, instead of Drd2, in PPX-elicited anti-depressive function in mice [33].

Mounting evidence reveals a complex crosstalk between the immune and nervous systems. DA and its receptor agonists modulate the immune system, in particular the innate immunity [34–36]. For example, DA and Drd1 signaling were reported to suppress NLRP3 inflammasome assembly in bone marrow-derived macrophages and control systemic inflammation [20]. Another study demonstrated that DA, via the stimulation of Drd5-ARRB2-PP2A signaling axis, blocked the TRAF6-mediated NF-κB pathway and inhibited systemic inflammation [9]. Interestingly, Drd2 null mice showed spontaneous inflammation in the brain, while *Drd1*^−/−^ mice did not [10], highlighting a role of astrocytic Drd2 signaling in inhibiting the transcription of proinflammatory cytokines physiologically. All these studies suggest that the mechanism responsible for DA and Drds agonists-elicited suppression of inflammation is cell- and receptor-dependent. In this study, we revealed that the Drds agonist PPX inhibited NLRP3 inflammasome activation via Drd3-dependent stimulation of autophagy flux in astrocytes. This provides a novel mechanism for the benefit of PPX treatment in PD because accumulative evidence from the patient with PD and animal models shows that NLRP3 inflammasome activation contributes to the pathogenesis of PD. Moreover, the DA neurons damage can be alleviated by NLRP3 inflammasome inhibitors [19–22]. Drd3 signaling may not serve as a critical controller of astrocytic inflammation in physiological condition since *Drd3* KO only resulted in mild alteration in the levels of proinflammatory mediators in the brain. However, Drd3 signaling is relevant for inflammation suppression in pathological conditions including PD [37]. Indeed, studies reported that activation of astrocytic Drd3 signaling may account for the anti-depressive function of PPX in PD-associated depression, which is also linked to neuroinflammatory responses [33, 38].

Autophagy is a lysosomal degradative process for the clearance of intracellular dysfunctional components including misfolded proteins and organelles. Dysregulation of autophagy participates in the pathogenesis of multiple neurodegenerative disorders including PD. Our previous study reported that Drd2 and Drd3 agonists promoted autophagy via a BECN1-dependent manner and had the potential to reduce α-syn accumulation in PD [15]. Other groups also found an enhanced autophagic flux in the brain of mice treated with PPX [39]. This is strengthened by a recent study reporting that PPX induced autophagy through a Drd3-dependent mechanism without suppression of protein synthesis [40]. This opens up a new approach for Drds agonists as autophagy inducers in the treatment of PD and other disorders. In this study, we presented the evidence that PPX also promoted the astrocytic autophagy flux via Drd3 signaling, in addition to its known effect on neuronal cells. Although researchers mostly focused on the relevance of autophagy in neurons, autophagy may also be important in maintaining astrocyte function, which plays a critical role in neurodegeneration via cell autonomous and non-cell autonomous machinery. A recent study identified impaired autophagy and progressive α-synuclein accumulation in PD patient-induced pluripotent stem cell-derived astrocytes and unraveled a crucial non-cell autonomous contribution of astrocytes during PD pathogenesis [41]. Furthermore, metabolic stresses differentially influence the autophagy in neurons and astrocytes, which coordinately maintain homeostasis in the brain [42]. Therefore, our results highlighted the role of autophagy in controlling NLRP3 inflammasome activation and IL-1β in astrocytes.

In summary, our study unveils a novel cellular and molecular mechanism that underlies the neuroprotection of the Drd3 agonist PPX in an inflammation-related mouse model of PD, and demonstrates the suppression of astrocytic NLRP3 inflammasome and IL-1β production by PPX through an autophagy-dependent mechanism.
